# Effects of a Stingless Bee Honey Mouth Rinse on Dental Plaque Accumulation: A Randomised Clinical Trial

**DOI:** 10.7759/cureus.86307

**Published:** 2025-06-18

**Authors:** Misya Humaira Reduan, Nor Suhaira Zulkaflee, Muhammad Annurdin Sabarudin, Nor Haliza Mat Baharin, Nik Madihah Nik Azis, Nur Ayman Abdul Hayei

**Affiliations:** 1 Faculty of Dentistry, Universiti Sains Islam Malaysia, Kuala Lumpur, MYS; 2 Periodontology, Faculty of Dentistry, Universiti Sains Islam Malaysia, Kuala Lumpur, MYS; 3 Restorative Dentistry, Universiti Kebangsaan Malaysia, Kuala Lumpur, MYS

**Keywords:** dental plaque, gingivitis, mouthrinse, plaque accumulation, stingless bee honey

## Abstract

Background

Mouth rinses are recommended as an adjunct to mechanical methods in oral hygiene procedures for the prevention and management of periodontal diseases. The currently used mouth rinses impose several side effects on patients, which include a burning sensation and altered taste. Alternatively, stingless bee honey (SBH) is a natural food with many health benefits, making it suitable as an active mouth rinse ingredient. Several characteristics, such as antibacterial, anti-inflammatory, and healing properties, make it a potential therapeutic agent in periodontal therapy as well as in the oral cavity. This study aimed to investigate the ability of SBH mouth rinses to reduce plaque accumulation and to assess patient acceptance of their use.

Methods

This is a randomised pilot clinical trial performed on volunteers (N = 30) with healthy gingiva and/or mild gingivitis, who were randomly prescribed SBH, chlorhexidine (CHX), or normal saline (NS) mouth rinses, without oral hygiene practices for three days. Plaque scores were recorded prior to the experiment by a single examiner, blinded to the treatment allocation. Plaque accumulation and patient perception were recorded post-intervention using the plaque score and visual analogue scale (VAS), respectively.

Results

At three days post-intervention, there was no significant difference in the plaque score of the SBH and NS groups (p > 0.05), compared to the baseline value. A significant decrease in the plaque score was found in the CHX group (p < 0.05), compared to the baseline. A significantly better taste and lower burning sensation were reported in the SBH group than in the NS and CHX groups, respectively.

Conclusions

The SBH mouth rinse did not promote plaque accumulation in the absence of oral hygiene and caused less burning sensation. CHX, however, has a greater ability to prevent plaque accumulation.

## Introduction

Mouth rinses are recommended as an adjunct to mechanical methods in oral hygiene procedures for the prevention and management of periodontal diseases. For more than 20 years, chlorhexidine (CHX) mouth rinses have been used to promote gingival health [1]. It has been well documented in the literature that the use of CHX mouth rinse is effective in combating gingivitis and dental plaque. It is considered the gold-standard antimicrobial agent [2]. However, despite its potent antimicrobial and anti-plaque properties, there have been various reports of undesirable adverse effects of CHX mouth rinse.

The prolonged use of CHX mouth rinse is limited by its local side effects. The adverse effects of CHX include extrinsic tooth staining, taste disturbance, and effects on the oral mucosa, such as soreness, irritation, mild desquamation, mucosal ulceration, and a general burning sensation [3]. Balagopal and Arjunkumar (2013) also reported that the side effects of CHX include brown discolouration of teeth, restorative materials, and the dorsum of the tongue; taste perturbation; dose-dependent oral mucosal erosion; and a bitter taste [4]. Mathur et al. (2011) added that it may also increase supragingival calculus formation due to its ability to precipitate salivary proteins on the tooth surface, increasing pellicle thickness and the precipitation of inorganic salts on the pellicle layer [2]. Therefore, there is a need to identify an alternative agent in mouth rinse that can also help in controlling plaque.

Stingless bees belong to the *Meliponini* subfamily, which is classified into two species: *Melipona* and *Trigona*, while common honeybees belong to the *Apis* genus. *Melipona* species are well distributed in tropical and subtropical areas, mainly Brazil, Indonesia, and Malaysia. *Trigona* species are generally found in tropical areas, with a predominance in Malaysia. Honeybees are found in Europe, Africa, the Middle East, and Asia. Stingless bee honey (SBH) consists of a great concentration and diversity of biologically active compounds, mainly flavonoids, phenolic acids, hydroxyls, and aromatics [5], making it a potentially emerging alternative agent in traditional medicine. It has greater antioxidant activity [6] and unique characteristics compared to other honeys, due to the cerumen pot-like storage that mixes honey with propolis. According to the Malaysian Agricultural Research and Development Institute (MARDI), stingless bees are easier to breed and are not choosy about where they produce their hives. They can produce new hives in abandoned hives, hollowed trees, or even buildings [6].

Active laboratory studies focused on antimicrobial [7], anti-inflammatory [8], and antidiabetic activities [9] were performed from 2014 to 2017. Its potential as a wound-healing [10] and anticancer [11] agent has since been explored. In vitro tests revealed that the antioxidant and biological activities of SBH are up to 45% greater than those of honey from *Apis mellifera* species [12]. SBH has recently been shown to have broad-spectrum antibacterial action against gram-positive and gram-negative bacteria, as well as fungi [13]. Current in vivo experimental studies have shown that SBH significantly protects against chronic lipopolysaccharide inflammation by reducing oxidative stress [14], reducing inflammation, and reducing dyslipidaemia-related lesions in dyslipidaemic rat colon epithelial cells [15]. SBH was found to be an effective antimicrobial and anti-inflammatory agent that could be used as an alternative agent in modern medicine.

Additionally, various studies have revealed that SBHs can treat burn wounds [16], chronic venous leg ulcers [17], and diabetic foot ulcers [18], indicating that SBHs have significant therapeutic value. It has also been proposed that SBH may lessen the severity of pulmonary symptoms in patients with infections caused by COVID-19, because of its anti-inflammatory effects [19]. This could be due to the synergistic effect of phenolic compounds, phenolic acids, and derivatives in honey, such as benzoic acid, caffeic acid, p-coumaric acid, ferulic acid, methyl syringate, sinapic acid, and 1,1-dimethylallyl caffeic acid ester [8]. It was reported that the high flavonoid content and low phenylethylamine content in SBH contribute to its antimicrobial activity [20].

In the oral cavity, natural honey was found to be effective against oral mucositis in cancer patients receiving radiotherapy [21], and was also found to enhance wound closure following tooth extraction in children [22]. Propolis, a hard, resinous compound produced by honey bees, was reported to be effective in reducing bad breath [23], suppressing cariogenic infection [24], and controlling gingival inflammation [25]. Recently, SBH from the genus *Trigona itama* was shown to exert a dose-dependent antiproliferative effect on in vitro oral squamous cell carcinoma (HSC-2) cell growth [26]. However, studies on the effects of SBH on the oral cavity are very limited, and no recommendations can be drawn. To date, no clinical study has investigated the efficacy of SBH in plaque control in the form of a mouth rinse. Hence, this study aimed to investigate the ability of SBHs to reduce plaque accumulation and to assess patients’ acceptance of the use of SBH mouth rinses. The specific objectives of this study were: (i) to investigate the anti-plaque activity of SBH in a clinical trial; (ii) to investigate patients’ acceptance of SBH mouth rinse; and (iii) to compare plaque levels after the use of SBH mouth rinse to those after the use of CHX mouth rinse.

This article was previously posted to the Research Square Preprint server on 29 July 2024.

## Materials and methods

Study design

This was a randomised, single-blinded, placebo-controlled clinical pilot study that was conducted at the Faculty of Dentistry, Universiti Sains Islam Malaysia (USIM), Kuala Lumpur, Malaysia. Ethical approval was obtained from the Research Ethics Committee, USIM (USIM/JKEP/2023-251). This clinical study followed the Consolidated Standards of Reporting Trials (CONSORT) statement and was registered on 24 January 2024 at ClinicalTrials.gov (No. NCT06223243).

Participants

Student volunteers from the Faculty of Dentistry, USIM, were invited to participate in the study. Written informed consent was obtained from the participants. The sample size for this pilot study was estimated to be 10 participants per arm, based on Whitehead et al. (2016) [27]. A total of 10 participants per group were recruited for the study based on the inclusion and exclusion criteria. The inclusion criteria were: (1) medically healthy dental students aged between 19 and 24 years; (2) healthy gingiva or localised gingivitis with the highest Basic Periodontal Examination (BPE) score of code 2; and (3) presence of at least 20 natural teeth. The exclusion criteria were: (1) patients with Type I or Type II diabetes mellitus; (2) any physical and/or mental disabilities that could impede the use of mouth rinse; (3) the use of orthodontic or prosthodontic appliances; (4) undergoing antibiotic or antimicrobial therapy within the past six months; (5) previous or current smokers; (6) saliva secretion rate outside the normal range for the population; (7) pregnancy or taking any hormone therapy; and (8) reported allergies to honey or bee stings.

Randomisation

Participants were randomly allocated to Group A, Group B, or Group C based on the order of recruitment. Randomisation was performed using Microsoft Excel software (Microsoft® Corp., Redmond, WA, USA). Predetermined recruitment numbers (1-30) were randomly allocated into Groups A, B, or C using computer-generated random numbers. The random allocation was generated and implemented by MHR and NSZ. Participants’ assignment and enrolment in the interventions were performed by MHR and NSZ based on this randomisation.

Blinding

This study was a single-blinded study in which each participant, and a single clinician who calculated the plaque scores, were blinded to the treatment assignment for the duration of the study. The clinician, NAAH, and participants were not involved in the intervention assignment.

Mouth rinse preparation

**Figure 1:**
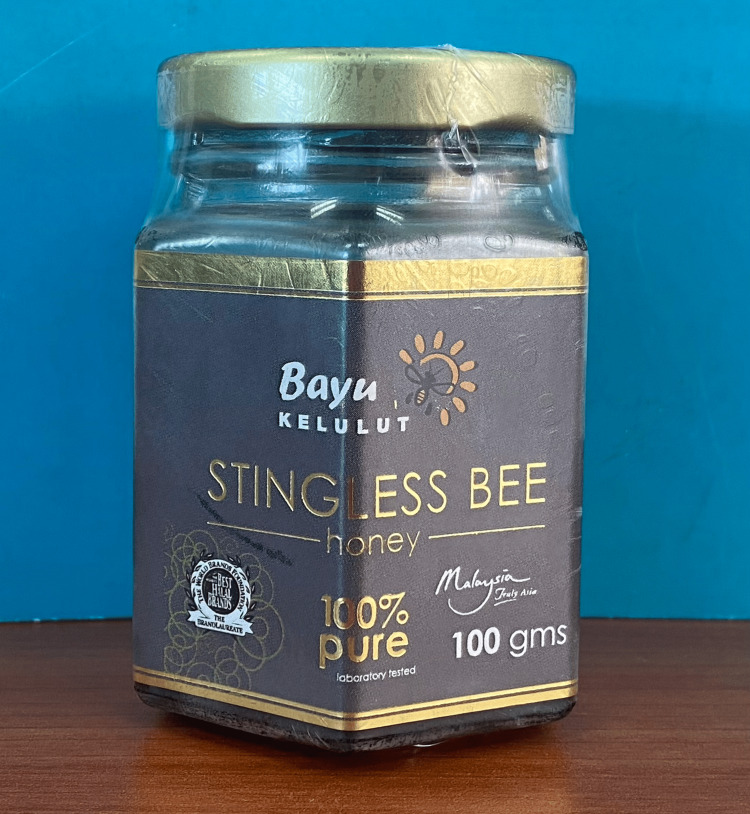
Stingless Bee Honey (Bayu Kelulut®)

**Figure 2:**
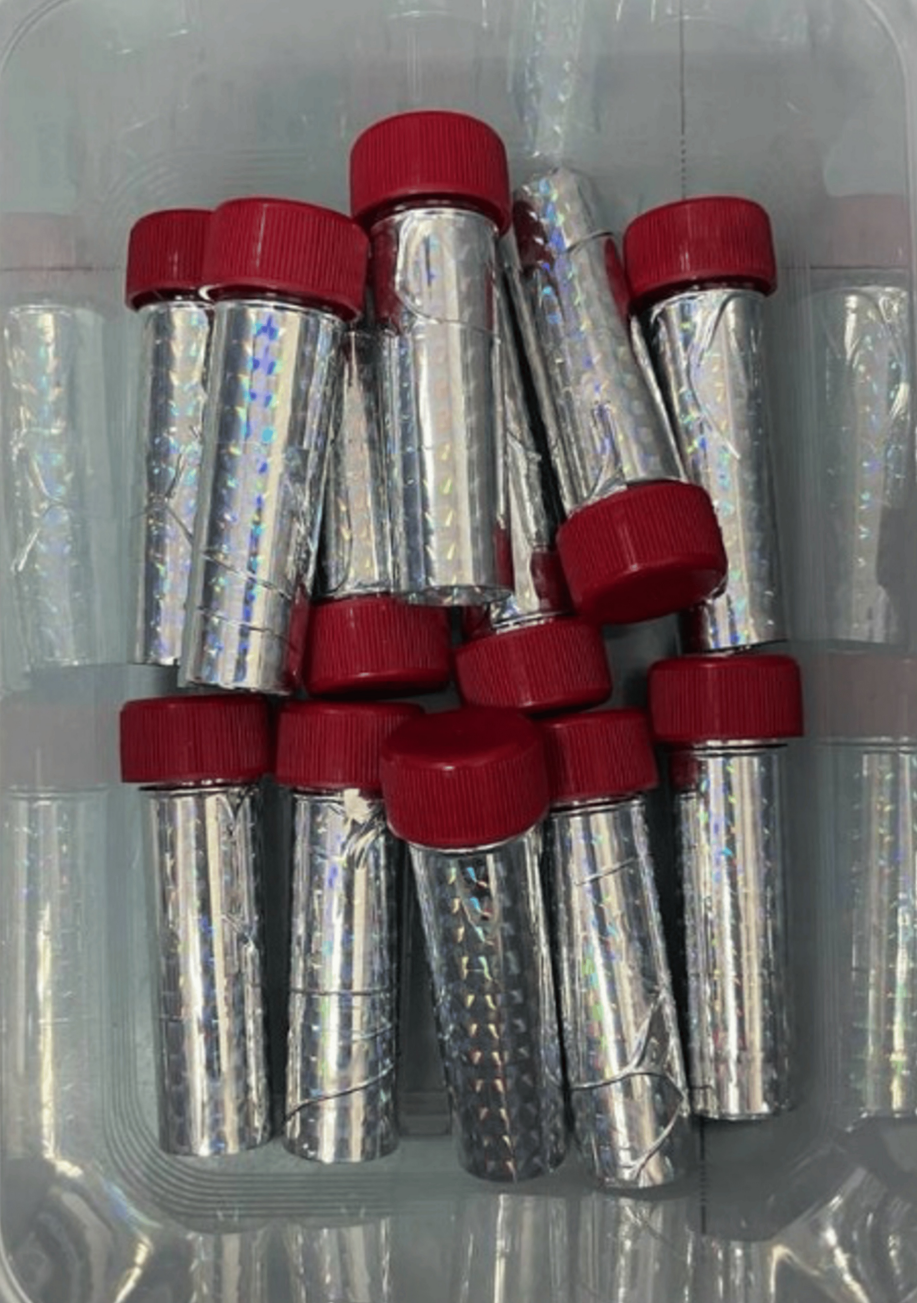
Sealed and Coded Mouth Rinse

Three different mouth rinse formulations were prepared in this study: (i) SBH (Bayu Kelulut®; Bayu Gagah Marketing (M) Sdn. Bhd., Kulim Hi-Tech Park, Malaysia) mouth rinse containing honey diluted with distilled water to a concentration of 20% (Figure 1), the minimum inhibitory concentration for oral pathogens as reported in a previous study [28]; (ii) 0.12% CHX (Oradex Antibacterial Mouthrinse; Cavico (M) Sdn. Bhd., Shah Alam, Malaysia, reg. no: MAL06011901XCZ); and (iii) placebo (negative control) containing 0.9% saline solution. These mouth rinse preparations were packed, sealed and coded in identical bottles before the start of the study, as shown in Figure 2. These formulations were mixed and kept in a refrigerator between 2°C and 8°C.

Intervention

Pre-intervention, plaque scores were taken at baseline by a single clinician (NAAH), a periodontist. This approach was used to reduce any variation in plaque score recording between different clinicians. Both the participants and the clinician were blinded to the intervention. After recording the plaque score, scaling and prophylaxis were performed by NAAH. This was to ensure a standardised level of hygiene among all the participants at the time of study commencement. Two dental students prescribed 90 mL of mouth rinse in a sealed bottle, following a randomised list. 

**Figure 3:**
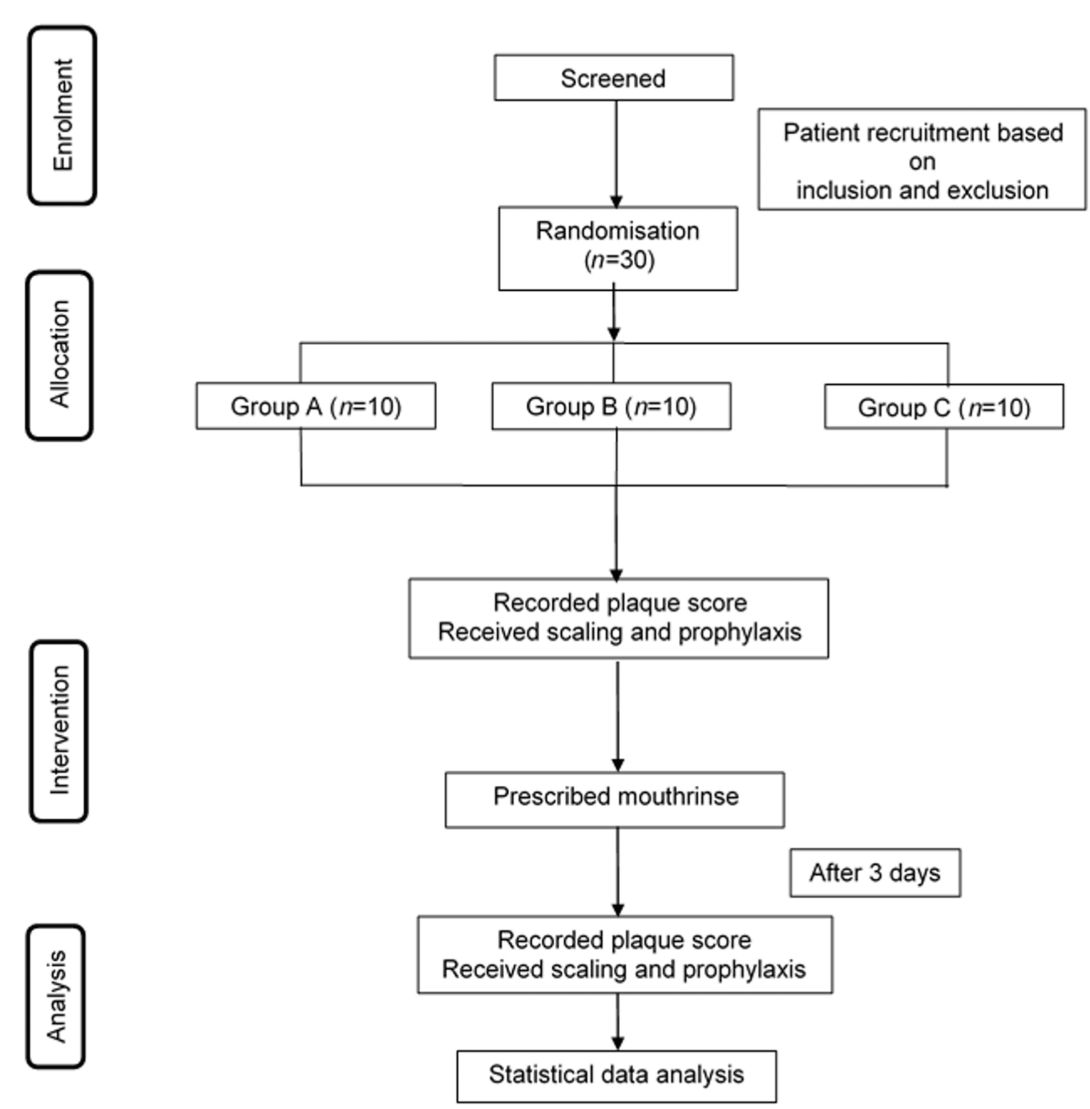
Flowchart of Study

Participants were instructed to refrain from all forms of tooth cleaning and to rinse 15 mL of mouthwash twice daily for two minutes. This was performed for three days. After three days, the plaque score was measured again by the same clinician (NAAH), and prophylaxis was given by NAAH, NHMB, or MAS. The visual analogue scale (VAS) score for patient perception was taken for taste, halitosis, burning sensation, and odour. The flow of the study is summarised in Figure 3.

Outcome measures

Patient Details

A self-report questionnaire for patients was administered on the first day of the experiment (T0), comprising questions on sociodemographic information (age, sex, and ethnicity), lifestyle habits, medical history, and dental history (see Appendix 1).

Plaque Score

Plaque accumulation was measured using the Plaque Index (PI), developed by O’Leary et al. (1972) [29], which measures the presence of supragingival plaque on four tooth surfaces. The plaque was scraped using a dental explorer. Plaque incidence in the oral cavity was expressed as an accurate percentage, and the presence (1) or absence (0) of plaque was noted in a simple chart.

Basic Periodontal Examination (BPE)

BPE of all the sextants was performed before and after the interventions. BPE was performed based on the guidelines of the British Society of Periodontology (BSP), published in 2019. A Community Periodontal Index and Treatment Needs (CPITN) probe was used for all procedures by a single clinician. There was one participant who had a BPE score of code 3 and was excluded. A full-mouth six-point charting was carried out, and an appointment was given for further periodontal therapy.

Visual Analogue Scale (VAS)

**Figure 4:**
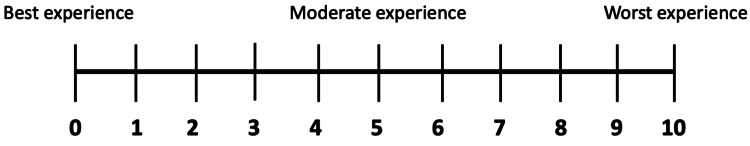
Visual Analogue Scale

Participants were asked about their acceptance or comfort with the mouthwash in terms of taste, halitosis, burning sensation, and odour. The level of acceptance or comfort was measured using a VAS (Figure 4). The VAS consisted of one line where the far-left end scale (0) indicated the best experience, while the far-right end (10) indicated the worst experience. Participants were instructed to choose any number between the two ends that best described the taste, halitosis, burning sensation, and odour they experienced during the intervention.

Data analysis

The data obtained were analysed using IBM SPSS Statistics for Windows, Version 27 (released 2020; IBM Corp., Armonk, NY, USA). The normality of the data was analysed using the Shapiro-Wilk test, and all the data were found to be normally distributed. Thus, intragroup comparisons of plaque scores (before and after treatment) were performed using paired sample t-tests. One-way analysis of variance (ANOVA), followed by Tukey’s post hoc test, was used to compare plaque scores between groups after the intervention and patient perceptions. All experimental results are presented as the mean ± standard deviation (SD). The mean values were considered statistically significant at p < 0.05.

## Results

Sociodemographic

A total of 32 participants were screened. One participant was excluded due to a BPE score greater than 2, and one participant was excluded due to the presence of oral mucosal inflammation. Both excluded participants were given appropriate advice and referred to specialists for further management. Table 1 summarises the sociodemographic characteristics of the participants in the CHX, normal saline (NS), and SBH groups. There were more females than males in this study, but the difference was not significant (p > 0.05). All of the participants were 21 to 22 years of age, had a tertiary education, were of Malay ethnicity, non-smokers, and did not wear dental appliances.

**Table 1:** Sociodemographic Characteristics of the Participants in the Three Groups CHX, chlorhexidine; NS, normal saline; SBH, stingless bee honey

Characteristics	CHX (n = 10)	NS (n = 10)	SBH (n = 10)	p-value
	n (%)	n (%)	n (%)	
Gender				
Male	1 (10)	3 (30)	6 (60)	0.256
Female	9 (90)	7 (70)	4 (40)	0.135
Age (years old)				
21-22	10 (33)	10 (33)	10 (33)	-
Education Level				
Tertiary	10 (33)	10 (33)	10 (33)	-
Ethnicity				
Malay	10 (33)	10 (33)	10 (33)	-
Medical Illness				
Asthma	1 (10)	0 (0)	0 (0)	-
None	9 (90)	10 (100)	10 (100)	-
Smoking				
None	10 (33)	10 (33)	10 (33)	-
Dental Appliance				
None	10 (33)	10 (33)	10 (33)	-

Plaque score

The mean plaque scores before the intervention (T0) were 34% (±13), 36% (±15), and 39% (±15) in the CHX, NS, and SBH groups, respectively (Table 2). After three days (T3) of cessation from any mechanical oral hygiene, a lower mean plaque score was recorded in the CHX group, 21% (±11), than in the NS and SBH groups, 56% (±17) and 45% (±15), respectively. However, compared to those at T0, none of the mouth rinses were significantly different at T3 (p > 0.05). A significant difference was reported when comparing CHX to NS and SBH (p < 0.05) at T3.

**Table 2:** Mean (SD) Plaque Score Between the Three Groups Before and After the Intervention Comparisons among the CHX, NS, and SBH groups after the intervention were performed using ANOVA and Tukey’s test. *Intragroup comparisons between baseline (T0) and post-intervention (T3) data were analysed using paired sample t-tests; **Comparison between CHX and NS indicated a statistically significant difference (p < 0.05); §Comparison between SBH and CHX indicated a statistically significant difference (p < 0.05). CHX, chlorhexidine; NS, normal saline; SBH, stingless bee honey; SD, standard deviation; ANOVA, analysis of variance

Group	Mean% (SD)		p-value	p-value
	T0	T3		
CHX (n = 10)	34 (13)	21 (11)	0.858*	<0.001**
NS (n = 10)	36 (15)	56 (17)	0.091	0.244
SBH (n = 10)	39 (15)	45 (15)	0.734	0.003§

Patient perception

**Figure 5:**
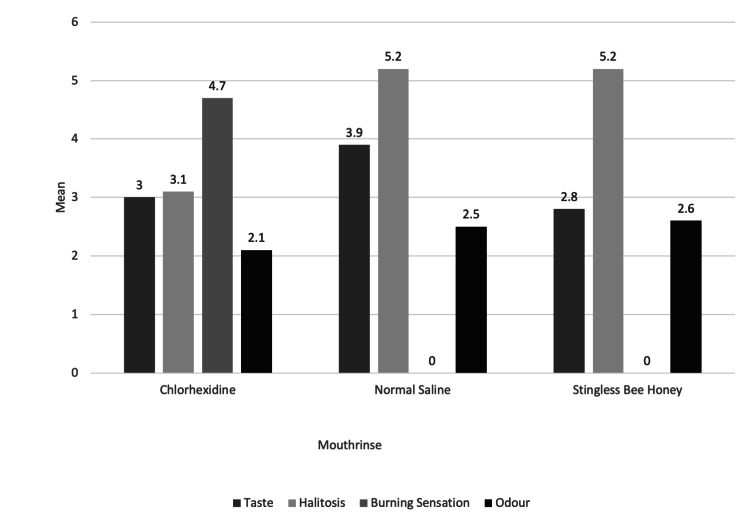
Patient Perception

The means (SDs) of patients’ perceptions are shown in Figure 5. Compared with both the CHX and NS mouth rinses, the SBH mouth rinse has a more acceptable taste: 1.9 (±1.10), 3.0 (±1.63), and 3.9 (±1.96), respectively. There was a significantly better taste perception after the use of SBH than after the use of NS (p < 0.05). CHX improved the perception of halitosis by a mean of 3.1% (±1.79) compared to NS, 5.2% (±1.48), and SBH, 5.2% (±2.66). There was no difference in the perception of halitosis improvement between the NS and SBH groups. In contrast, the CHX group had a greater perception of burning sensation, 4.7 (±2.31), than did the NS and SBH groups. A significantly greater burning sensation was reported in the CHX group than in the NS and SBH groups (p > 0.05). However, for the perception of odour, there was no difference among the three groups.

## Discussion

SBH has been shown to have antibacterial properties [30], validating its use in traditional medicine and its rise in modern medicine. This is attributed to several variables, including its hydrogen peroxide content, low pH, and high osmolarity [31]. In this study, there was no significant increase in plaque scores after three days of oral hygiene or the use of an SBH mouthwash, further supporting the previous statement. The antimicrobial effect of SBH, due to its phenolic compound content [8,20], could have prevented bacterial aggregation and adhesion during plaque formation [32]. The formation of mature dental plaque requires primary and secondary attachment of bacteria to the tooth surface. The absence of primary and secondary layers of bacteria prevented the formation of a structured dental plaque [32]. In addition, the alkaline environment in SBH is unfavourable for cariogenic bacteria [33].

Honey was shown to inhibit the activity of *Porphyromonas gingivalis* [34], a potent periopathogen that is consistently associated with periodontitis [35]. Furthermore, it was reported that SBH has the ability to inhibit the growth of gram-positive and gram-negative bacteria [7], which are found in supra- and subgingival dental plaque [36]. The hydrogen peroxide content in SBH enhances the production of cytokines in the inflammatory response to kill bacteria [16]. In addition, the phytochemicals in SBH, such as flavonoids, phenolic substances, and antibacterial peptides, directly inhibit phagocytosis and prevent superoxide free radicals from damaging tissues. The acidity of SBH also suppresses the majority of bacteria that grow at pH values between 7.2 and 7.4 [37].

On the other hand, CHX contains bisbiguanide, a chemical compound that exerts both bacteriostatic and bactericidal effects. The use of CHX mouthwash effectively reduces bacteria [38]. Therefore, the formulation of SBH can be improved by increasing the phenolic compounds and their derivatives, while maintaining its taste, to provide enhanced antibacterial effects.

In terms of taste, SBH had a significantly better score than did NS. Various sugars, including fructose, glucose, and sucrose, have been found in the contents of SBH [39]. This gave the SBH mouthwash a sweet taste, which is universally preferable [40]. There was a risk of dental caries development due to the presence of fermentable sugars in SBH; therefore, the ‘no oral hygiene’ period was limited to three days. The earliest sign of dental caries is enamel demineralisation, which takes at least one week to occur [41]. When the cariogenic challenge is removed, remineralisation occurs completely within a few weeks [41]. Fluoride application has also been documented as an effective remineralisation technique [42]. Despite honey’s sweet taste, honey has the ability to inhibit the growth and acid production of cariogenic bacteria, including *Streptococcus mutans* [43].

CHX had a significantly greater effect on burning sensation than both NS and SBH. This is a known side effect of CHX, which is unacceptable for some patients [44]. There are no reports of a burning sensation for SBH, which could be because SBH has a nontoxic and anti-inflammatory effect [31]. Various studies have reported that burning sensations are a limitation of CHX, along with taste disturbance, tongue and tooth discolouration, oral mucosal soreness, and supragingival calculus formation [45].

No differences were found in the perception of halitosis. Although both CHX and SBH have been shown to have antibacterial activity, their ability to reduce malodor was not exhibited in this study. This is most likely due to the short intervention period, and considering that this test is solely based on the patient’s self-assessment. There were also no differences in the perception of odour among the three groups. This could be due to the varying preferences for smell between different individuals, making it complex to determine their preferences for different odours [46].

Limitations

This was a single-centre intervention performed with a small number of participants. In addition, comparisons of patient perceptions between different mouth rinses were not performed because each participant used only one type of mouthwash. Ideally, testing different mouth rinses on similar participants, with a washout period, would be ideal to avoid any carryover effects [47].

Recommendations

Although less potent than CHX, SBH has been shown to be effective at preventing a significant increase in plaque accumulation. In addition to its antibacterial properties, it also has an acceptable taste and no burning sensation. The addition of fluoride to the SBH mouthwash formulation is recommended to prevent dental caries. Further research, investigating the potential of SBH in adjunctive periodontal therapy with a larger sample size and a longer follow-up period, such as in a multicentre study, is warranted. In vitro testing against periodontal pathogens, and maximising the antibacterial components in the mouthwash, can provide better verification of the properties of SBH and its applications in oral health care. Finally, different mouth rinses may also be used on the same patient in future studies to compare the effects. However, this would take longer, as a washout period is needed to prevent any residual effects or perceptions that could alter the results. To avoid tooth demineralisation, participants should be screened for caries risk and receive fluoride therapy post-intervention.

## Conclusions

SBH mouthwash was able to prevent a significant increase in plaque accumulation in the absence of oral hygiene, with no burning sensation and an acceptable taste. However, CHX has a better ability to prevent plaque accumulation. Further study of the promising antiplaque properties of SBH, aimed at using its products in complementary dental therapy, is recommended.

## Appendices

Appendix 1: Questionnaire form

The objectives of this study are as follows:

a) To evaluate patients' subjective preferences for scaling with narrow probe-shaped EMS Perio Slim PS tips compared to universal tips based on pain perception using the Visual Analogue Scale (VAS).

b) To evaluate clinicians’ perception and preference for the different scaler tip designs.

All the information recorded from this questionnaire will be kept confidential. The data collected will be used to improve the quality of patient’s care. Kindly answer all questions. Thank you.

Please tick √ in the appropriate boxes.

Demographic data

a) Age (as of 1/1/17): ___________

b) Ethnicity

  ☐ Malay

  ☐ Chinese

  ☐ Indian

  ☐ Others, please state _______,

c) Gender:

  ☐ Male

  ☐ Female

Lifestyle habits

1) Level of education:

  ☐ Primary

  ☐ Secondary

  ☐ Tertiary

  ☐ Others, please state _______

2) Dental visits:

  ☐ Regular

   ☐ ≥2 times/year

   ☐ <2 times/year

  ☐ Irregular

3) Oral hygiene habits:

  Brushing

   ☐ <1x/ day

   ☐ 1x/ day

   ☐ >1x/ day

  Interdental cleaning

   ☐ Flossing

   ☐ Tooth pick

   ☐ Interdental brush

  ☐ Mouth rinse
